# Supplementary material for: Fabrication and Characterization of Bioresorbable Drug-coated Porous Scaffolds for Vascular Tissue Engineering
Source: Materials (Basel). 2019 May 2;12(9):1438. doi: 10.3390/ma12091438 (PMC6539797; doi:10.3390/ma12091438)
Supplement: Supplementary file 1 [file materials-12-01438-s001.pdf]

Supplementary Information

Fabrication and Characterization of Bioresorbable Drug-coated Porous Scaffolds for Vascular Tissue Engineering

Jueun Kim [1], Su A. Park [2], Jei Kim [3] and Jaejong Lee [1,*]

[1] Department of Nano Manufacturing Technology, Nano-Convergence Mechanical Systems Research Division, Korea Institute of Machinery & Materials (KIMM), 156 Gajeongbuk-Ro, Yuseong-Gu, Daejeon 34103, Korea; jekim@kimm.re.kr
[2] Department of Nature-inspired Nano Convergence Systems, Nano-Convergence Mechanical Systems Research Division, Korea Institute of Machinery & Materials (KIMM), 156 Gajeongbuk-Ro, Yuseong-Gu, Daejeon 34103, Korea; psa@kimm.re.kr
[3] Department of Neurology, Chungnam National University Hospital, 282 Munhwa-ro, Jung-gu, Daejeon 35015, Korea; jeikim@cnu.ac.kr
* Correspondence: jjlee@kimm.re.kr; Tel.: +82-42-868-7145

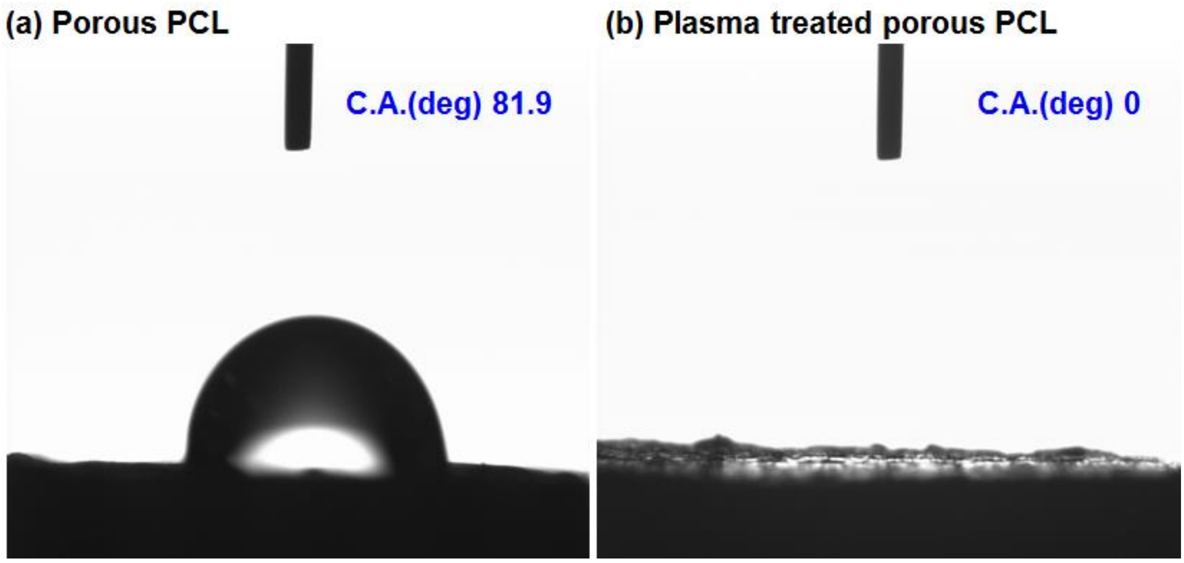

Figure S1. Water contact angle measurement before (**a**) and after plasma treatment (**b**).

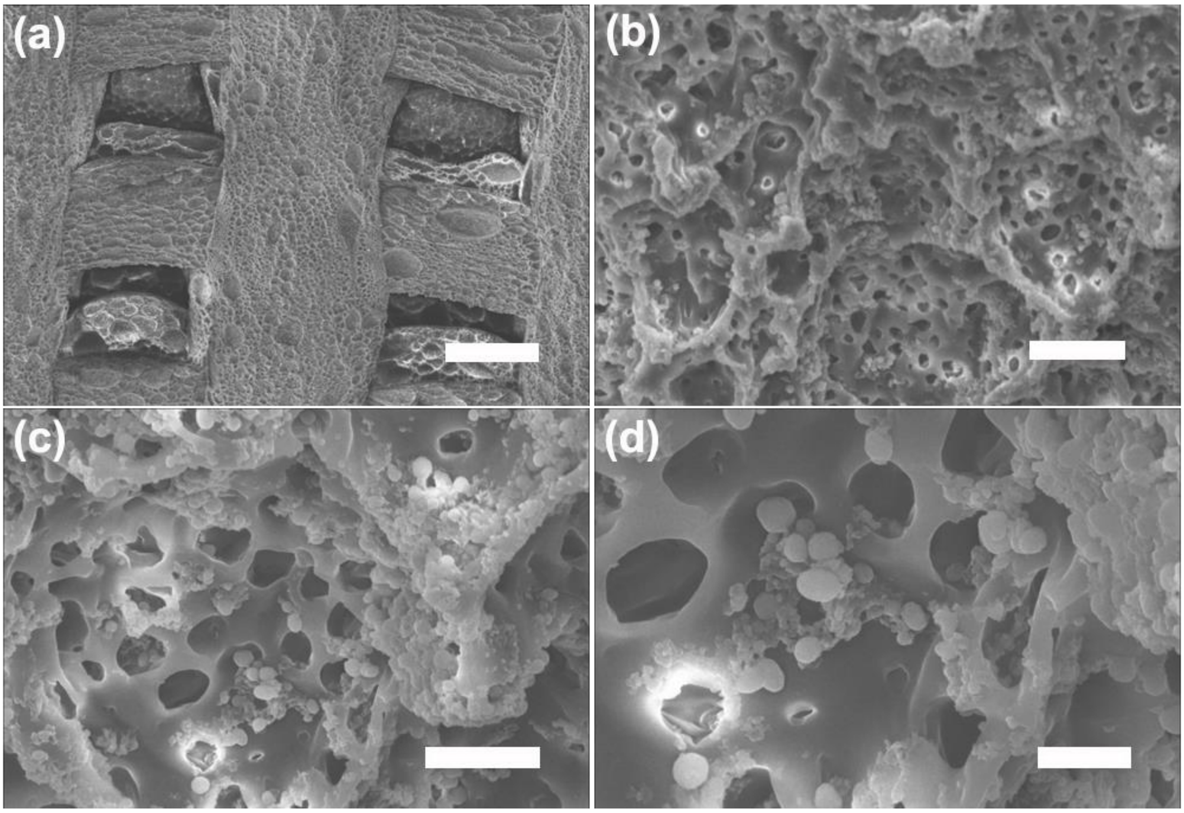

Figure S2. SEM image of the particles formed on the surface of the drug-coated porous PCL scaffold. Scale bars: 200 µm (**a**), 10 µm (**b**), 5 µm (**c**) and 2 µm (**d**).

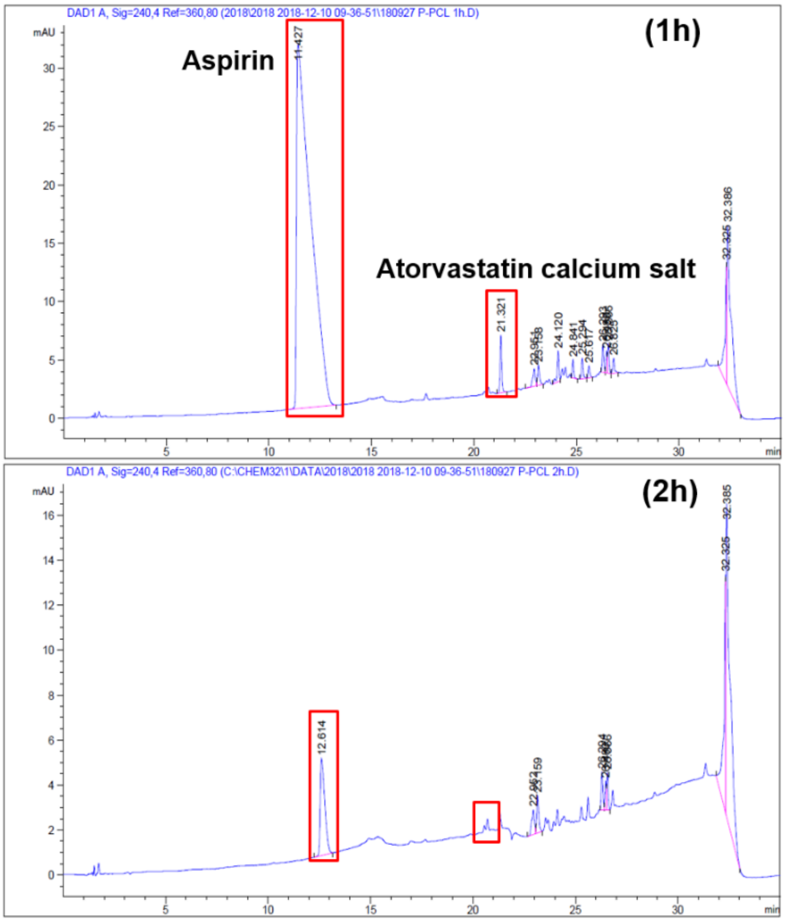

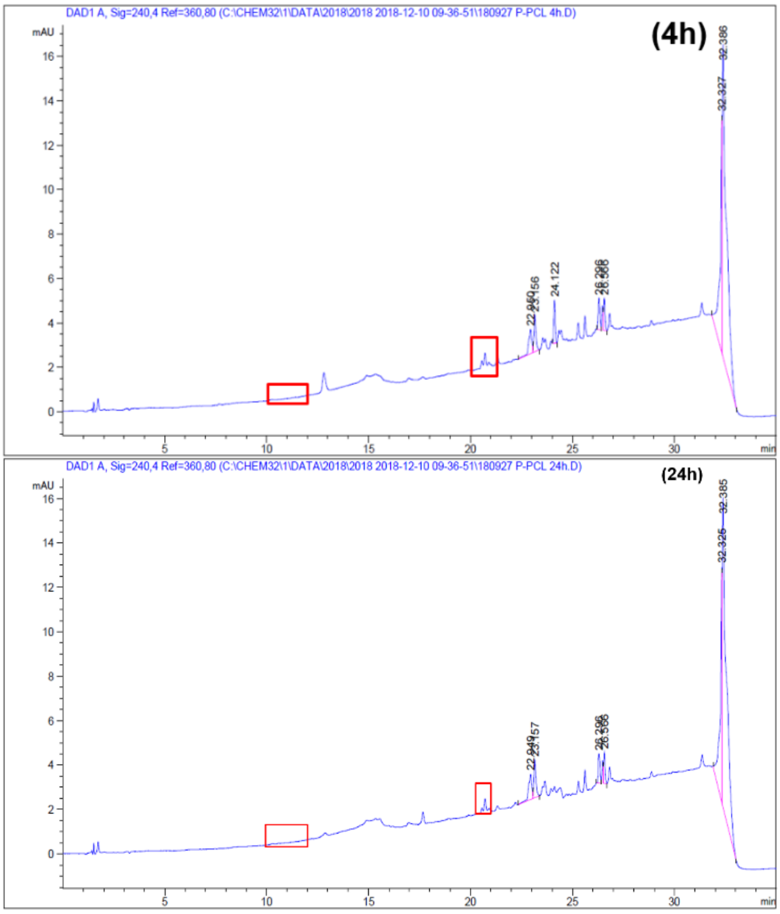

Figure S3. Drug content of the drug-coated porous PCL scaffold after drug release for 1, 2, 4 and 24 h.

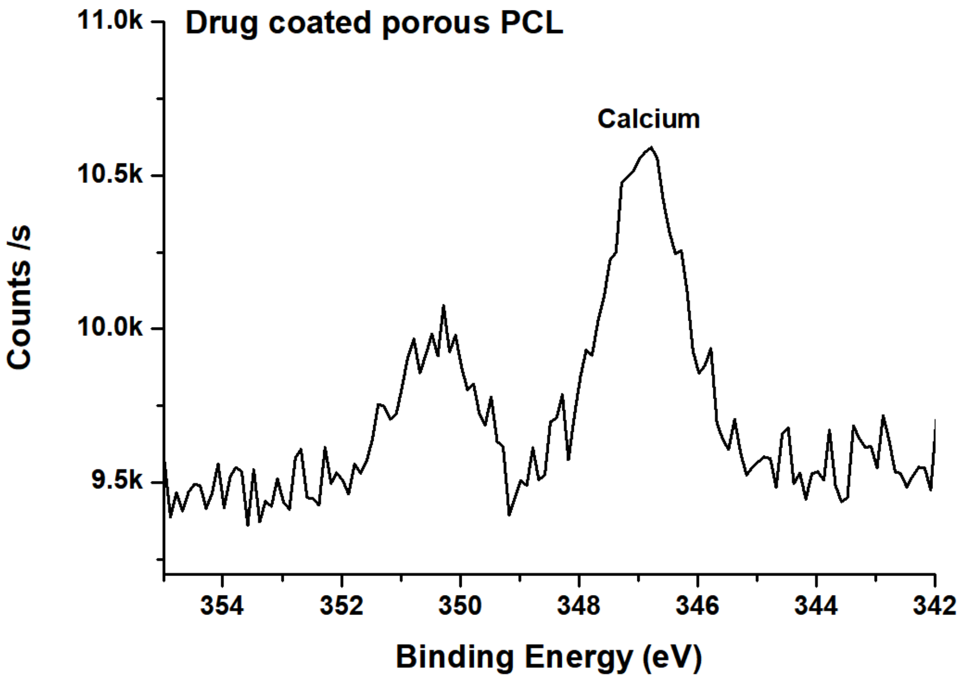

Figure S4. XPS analysis of the high-resolution Ca2p peak at 347 eV observed only in the spectrum of the drug-coated porous PCL scaffold.
